# Patent foramen ovale is associated with white matter hyperintensities in young adults: a case-control study

**DOI:** 10.3389/fneur.2026.1906472

**Published:** 2026-09-03

**Authors:** Changjiang Chen, Dan Gu, Jiayi Lu, Chuanling Wang, Zhiyou Cai

**Affiliations:** 1 The People‘s Hospital of Tongnan District, Chongqing City, Chongqing Tongnan District People’s Hospital, Chongqing, China; 2 Department of Neurology, People’s Hospital of Chongqing Liangjiang New Area, Liangjiang Hospital of Chongqing Medical University, Chongqing, China; 3 Chongqing Medical University, Chongqing, China; 4 Chongqing Key Laboratory of Neurodegenerative Diseases, Chongqing, China; 5 Department of Pathophysiology, Chongqing Medical University, Chongqing, China

**Keywords:** PFO (patent foramen ovale), right-to-left shunt (RLS), risk factors, white matter hyper intensity, young adults

## Abstract

**Objective:**

To investigate the association between patent foramen ovale (PFO) and white matter hyperintensities (WMH) in young adults and to assess whether PFO may be associated with WMH in this population.

**Methods:**

A multicenter retrospective case-control study was conducted. 120 young adult PFO patients aged 18–55 years diagnosed between October 2025 and March 2026 at two tertiary general hospitals in Chongqing, China, formed the case group. 121 age- and sex-matched controls were selected from the same period. The control group consisted of individuals who underwent cranial MRI and PFO-related examinations during the same period for similar clinical indications (e.g., headache, dizziness, or cryptogenic stroke/TIA workup) but were confirmed to have no PFO by transesophageal echocardiography. These participants were not necessarily “healthy” in the community sense but served as a clinical control group without the exposure of interest (PFO). Clinical data were collected for both groups. Transesophageal echocardiography (TEE) and cranial magnetic resonance imaging (MRI) were performed to measure PFO diameter and assess right-to-left shunt (RLS) severity. WMH severity was graded using the Fazekas scale. The chi-square test and Mann–Whitney *U* test were used to compare WMH grading differences between groups. A partial correlation analysis examined the relationship between PFO indicators and WMH grading. A multinomial logistic regression model was constructed to evaluate the association between PFO and WMH in young adults. Exclusion criteria included hypertension, diabetes, cerebrovascular disease, head and neck arterial stenosis ≥50%, and other cardiac structural abnormalities. Clinical indications for cranial MRI and PFO-related examinations in both groups included headache/migraine, dizziness, cryptogenic stroke/TIA, and other neurological symptoms, with distributions balanced between the PFO and control groups through the matching process.

**Results:**

WMH grade was significantly higher in the PFO group than in the control group (*p* < 0.001). PFO diameter and RLS grade were positively correlated with WMH grade (*p* < 0.05). After adjusting for confounding factors including age and sex, PFO diameter remained significantly associated with WMH grade in young adults.

**Conclusion:**

These findings suggest that PFO diameter may be associated with WMH severity in young adults. The underlying mechanisms remain to be elucidated and warrant further investigation.

## 1 Introduction

White matter hyperintensity (WMH) refers to areas of the brain’s white matter that appear as high signal intensity on T2-weighted magnetic resonance imaging (MRI) or fluid-attenuated inversion recovery (FLAIR) sequences. It primarily reflects pathological changes, including cerebral microvascular disease, demyelination, and gliosis. The severity of WMH correlates with age and traditional vascular risk factors, including hypertension and diabetes (1–3). However, in some patients, significant white matter lesions persist even after controlling these risk factors, suggesting the potential existence of other underlying pathogenic mechanisms (4).

Patent foramen ovale (PFO) is a common congenital structural heart abnormality, with a prevalence as high as 79% among patients with cryptogenic stroke and migraine (5). As the primary cause of right-to-left shunt (RLS), PFO typically presents no significant clinical symptoms in healthy individuals and has thus long been considered a benign anatomical variant (6, 7). However, recent studies have confirmed that occult PFO can enable microemboli from the venous system to bypass pulmonary circulation and directly enter cerebral circulation through an anomalous embolization mechanism. This causes chronic damage to cerebral microvasculature while simultaneously affecting cerebral perfusion status, making it a potential underlying cause of occult cerebrovascular injury in young adults. It also provides crucial foundational evidence for its pathological association with WMH (4, 8, 9).

The association between PFO and WMH has become a research hotspot in cerebrovascular disease. Current single-center studies confirm that young PFO patients exhibit significantly higher WMH detection rates and lesion burdens compared to healthy individuals. Furthermore, PFO diameter and RLS classification correlate positively with WMH severity, indicating a clear quantitative association between the two (8). Concurrently, the cerebral circulatory hemodynamic abnormalities induced by RLS reduce effective perfusion in white matter regions, exacerbating myelin loss and axonal damage, ultimately contributing to the development and progression of WMH (10). At present, there remains a lack of multicenter evidence-based validation regarding the independent pathogenic role of PFO in WMH among young adults, and the specific mechanisms underlying their association, as well as the clinical value of intervention, remain unclear. This study systematically investigates the quantitative relationship between PFO diameter, RLS classification, and WMH severity in young PFO patients, thereby providing evidence-based medical support for etiological screening, risk stratification, and early intervention of WMH in young adults.

## 2 Methods

### 2.1 Study population

**Figure 1:**
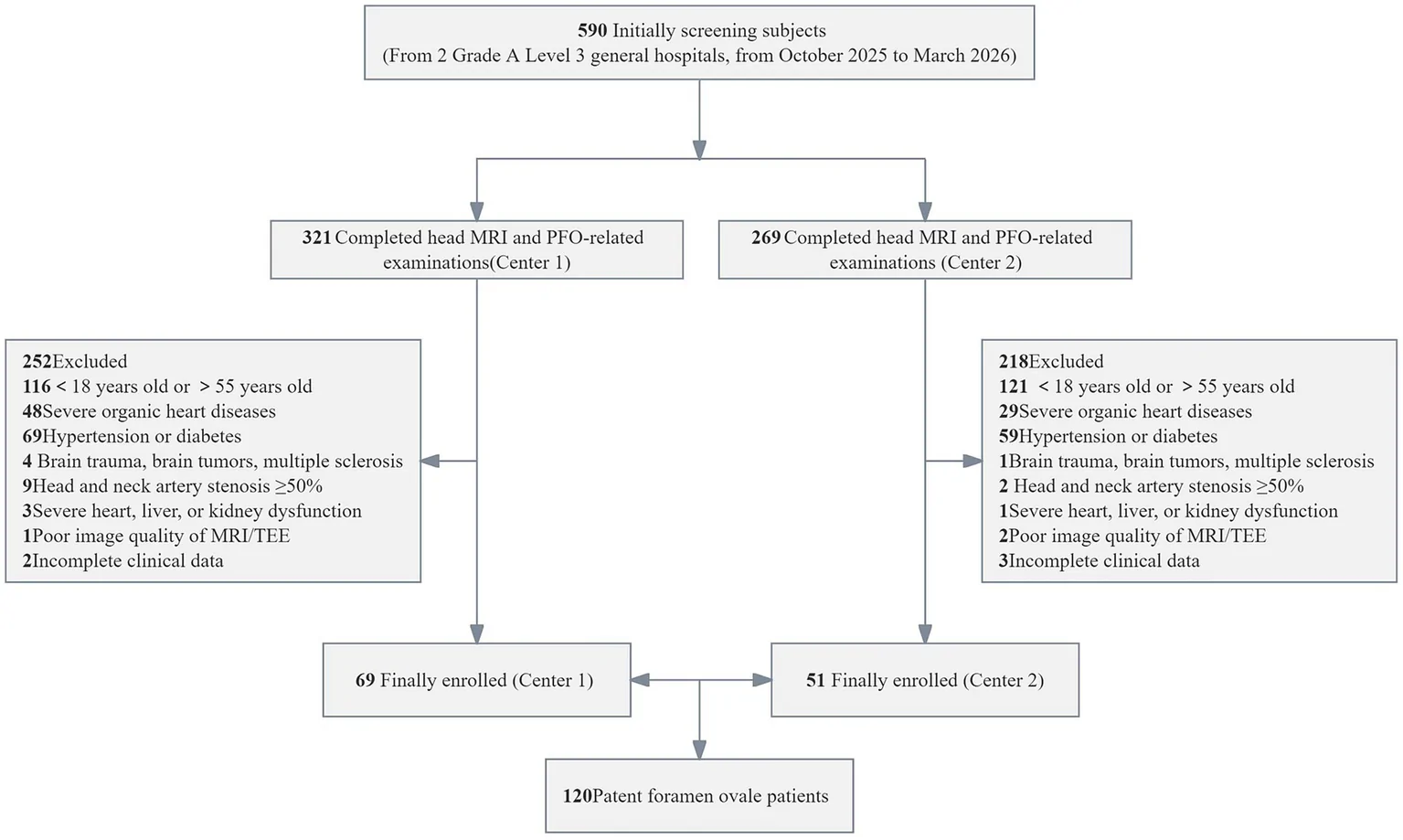
Flowchart of patient enrollment process.

This study employed a multicenter, retrospective case–control design and was conducted in collaboration with two tertiary general hospitals in Chongqing, China. A total of 241 young adults who underwent cranial MRI and PFO-related examinations at each center between October 2025 and March 2026 were enrolled. A 1:1 matched case–control design was employed, comprising 120 cases and 121 controls. The control group was strictly matched with the case group based on age, sex, and baseline characteristics. Inclusion criteria: (1) young adults aged 18 ~ 55 years; (2) Completion of transesophageal echocardiography (TEE); the case group was definitively diagnosed with PFO via TEE (11), while the control group consisted of individuals without PFO; (3) Completion of cranial MRI, with imaging sequences including T2-weighted and FLAIR sequences; (4) Complete clinical data. Exclusion criteria: (1) Cardiac structural abnormalities other than PFO; (2) History of other diseases that can clearly cause white matter lesions, such as traumatic brain injury, brain tumors, or multiple sclerosis; (3) History of hypertension, diabetes, or cerebrovascular disease; (4) Head and neck arterial stenosis ≥50%; (5) Severe hepatic or renal insufficiency and dysfunction of vital organs; (6) Poor quality of imaging data, such as cranial MRI or TEE. The clinical indications for cranial MRI and PFO-related examinations in both groups included evaluation of headache, migraine, dizziness, cryptogenic stroke/transient ischemic attack (TIA), syncope, or screening for cardiogenic embolism in patients with unexplained neurological symptoms. The distributions of these indications were balanced between the PFO and control groups through the matching process. This information is provided to address potential selection bias, as patients evaluated for different indications may have varying baseline WMH risk (Figure 1).

This study was conducted in accordance with the principles of the Declaration of Helsinki. The research protocol has been approved by the Medical Ethics Committee of the lead institution, People’s Hospital of Chongqing Liangjiang New Area (Approval No.: 2026015), and all participating institutions have endorsed the committee’s decision. As this study involves retrospective data analysis, the Ethics Review Committee has approved a waiver of written informed consent.

### 2.2 Data collection

A standardized, anonymized data collection form was developed to record the participants’ general demographic information, clinical data, and imaging findings. Two researchers performed data entry.

During transesophageal echocardiography (TEE), an experienced sonographer measured the maximum diameter of the PFO using an electronic caliper at the end of ventricular diastole. The classification and grading of RLS were confirmed using transcranial Doppler (TCD) and the bubble test during both rest and the Valsalva maneuver. In this study, TEE was used for anatomical diagnosis of PFO and measurement of its maximum diameter, as well as for direct visualization of microbubbles in the left atrium during contrast echocardiography. TCD with a bubble test was used to functionally grade RLS severity based on the number of microbubbles detected in the middle cerebral artery. These two modalities were used complementarily: TEE provided anatomical and structural information, while TCD provided quantitative functional assessment of shunt burden. The RLS grading results were determined by consensus between the TEE and TCD findings. Provoked RLS occurs only after the Valsalva maneuver, whereas persistent RLS is present even at rest. The severity of RLS was graded based on the number of microbubbles detected in the left atrium, using a refined four-tier classification system in accordance with authoritative domestic guidelines, while also aligning with the internationally accepted three-tier classification system. Specifically: (1) Grade 1 (Mild): 1–10 bubbles; (2) Grade 2 (Moderate): 11–25 bubbles; (3) Grade 3 (Severe): >25 bubbles, presenting as localized curtain-like imaging (classified as International Grade 3); (4) Grade 4 (Extremely Severe): bubbles cannot be counted, presenting as full-screen curtain-like imaging (classified as International Grade 3) (12). RLS grading results are based on the highest degree of microbubble enhancement observed after the Valsalva maneuver. All examinations were performed independently and in a blinded manner by two senior radiologists; any discrepancies were resolved by consensus.

WMH assessment was performed using a 3.0 T MRI scanner, with standard scanning sequences including axial T1WI, T2WI, and T2-FLAIR sequences covering the entire brain. WMH grading was performed using the internationally recognized Fazekas visual rating scale (13). Two senior neurologists, blinded to PFO status, independently assessed periventricular hyperintensity (PVH) and deep white matter hyperintensity (DWMH). Grading ranges from 0 to 3: (1) Grade 0: neither PVH nor DWMH is present; (2) Grade 1: PVH appears as a cap-like or fine-line pattern; DWMH appears as punctate lesions; (3) Grade 2: PVH appears as a smooth ring; DWMH shows lesions beginning to merge; (4) Grade 3: PVH extends irregularly into the deep white matter; DWMH appears as large, merged lesions. Any discrepancies in grading were resolved through discussion to reach a consensus. Inter-rater agreement was assessed using the Kappa test, with a Kappa value ≥0.80 considered to indicate good agreement. The primary focus of this study was to investigate the association between PFO and WMH severity as a risk factor analysis, rather than to provide a comprehensive characterization of all cerebral small vessel disease (CSVD) imaging markers. Therefore, the MRI assessment was deliberately centered on WMH grading using the validated Fazekas scale, which is the most widely accepted and clinically practical tool for WMH severity assessment. Other CSVD markers—including lacunes, enlarged perivascular spaces, cerebral microbleeds, and detailed lesion distribution analysis (e.g., cortical/juxtacortical involvement or posterior circulation patterns)—were not systematically evaluated, as they were beyond the scope of this study’s primary objective.

### 2.3 Statistical analysis

All statistical analyses were performed using SPSS 29.0 and R 4.4.3 software. Continuous variables that followed a normal distribution were expressed as mean ± standard deviation (x̅ ± s), and comparisons between groups were performed using the independent samples *t*-test; those that did not follow a normal distribution were expressed as median (interquartile range) (IQR), and comparisons between groups were performed using the Mann–Whitney *U* test. Categorical data are presented as counts (percentages), and comparisons between groups were performed using the chi-square (χ^2^) test. Partial correlation analysis, adjusted for age and sex, was used to explore the associations between PFO diameter, RLS grade, and WMH grade. With WMH grade as the dependent variable, variables with *p* < 0.05 in univariate analysis were included in a multivariate model. A multinomial logistic regression model was used to identify independent predictors of WMH severity, as the proportional odds assumption for the ordered logistic regression was violated (parallel lines test, *p* < 0.05). We proceeded with multinomial logistic regression analysis. To address the high collinearity among PFO-related indicators (PFO diameter, RLS grade, and RLS type), a forward stepwise selection method (entry criterion *p* < 0.05, removal criterion *p* > 0.10) was applied to identify the most robust independent predictor(s) for WMH severity. All tests were two-sided, and a *p*-value <0.05 was considered statistically significant.

## 3 Results

### 3.1 Comparison of demographic characteristics between the two groups

This study included 120 patients in the PFO group and 121 in the control group. There were no statistically significant differences between the two groups in terms of age [45 (37, 51) years vs. 47 (39, 52) years, *p* = 0.682] or gender distribution [38.3% male vs. 43.0%, *p* = 0.463], indicating good comparability (Table 1). The median PFO diameter in the PFO group was 10.0 (7.8, 12.1) mm. The distribution of RLS grades showed that the PFO group was predominantly Grade 1 (24.2%) and Grade 3 (31.4%), with relatively lower proportions of Grade 2 (22.5%) and Grade 4 (21.6%); the RLS subtypes were predominantly induced shunts (71.7%), with persistent shunts accounting for 28.3%.

**Table 1:** Comparison of baseline characteristics between the PFO group and the control group.

Variables	PFO group (*n* = 120)	Control group (*n* = 121)	*Z/χ^2^*	*p* value
Age (years)	45 (37, 51)	47 (39, 52)	−0.410^a^	0.682
Sex [*n* (%), male]	46 (38.3%)	52 (43.0%)	0.538^b^	0.463
Hypertension [*n* (%)]	0 (0.0%)	0 (0.0%)	—	1.000
DM [*n* (%)]	0 (0.0%)	0 (0.0%)	—	1.000
CHD [*n* (%)]	0 (0.0%)	0 (0.0%)	—	1.000
PFO diameter (mm)	10.0 (7.8, 12.1)	—		
RLS grade [*n* (%)]				
Grade 1	29 (24.2%)	—		
Grade 2	27 (22.5%)	—		
Grade 3	38 (31.7%)	—		
Grade 4	26 (21.6%)	—		
RLS type [*n* (%)]				
Provoked	86 (71.7%)	—		
Persistent	34 (28.3%)	—		

PFO, patent foramen ovale; DM, diabetes mellitus; CHD, coronary heart disease; IQR, interquartile range; RLS, right-to-left shunt.

Data are presented as median (IQR) or *n* (%). a, Mann–Whitney *U* test; b, Chi-square test. Since hypertension, diabetes, and coronary heart disease were absent in both groups, the chi-square test statistic was not calculated, and the P value is considered 1.000. Control group: Individuals without PFO, matched for age and sex from the same clinical population.

### 3.2 Comparison of WMH grading between the two groups

**Figure 2:**
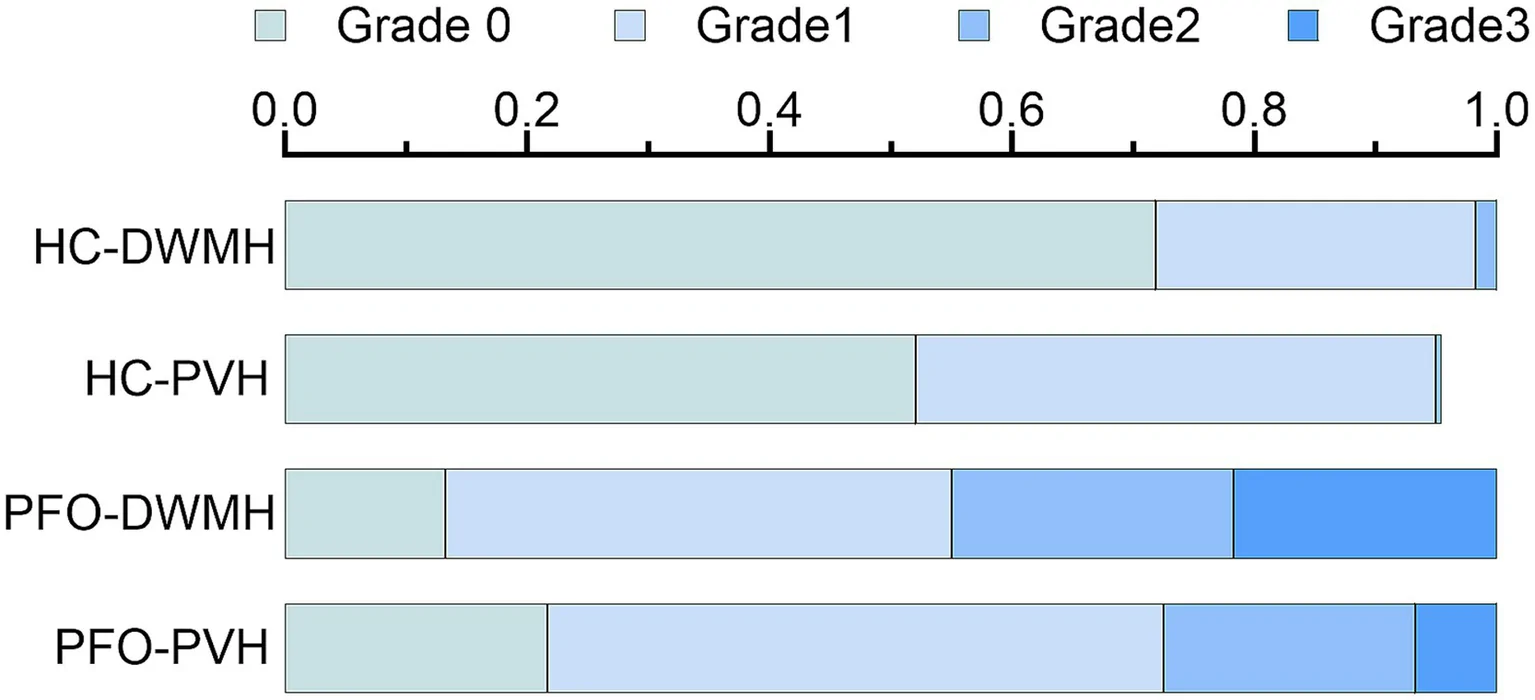
Distribution of white matter hyperintensity (WMH) grades in the patent foramen ovale (PFO) group and control group. The stacked bar chart shows the proportions of periventricular hyperintensity (PVH) and deep white matter hyperintensity (DWMH) across grades 0–3 in the two groups. Compared with the control group, the PFO group had significantly lower proportions of grade 0 WMH and significantly higher proportions of grades 1–3 WMH for both PVH and DWMH, with statistically significant between-group differences (all *p* < 0.05).

Compared with the control group, the PFO group had a significantly lower proportion of Grade 0 PVH and DWMH, and a significantly higher proportion of Grades 1–3; the differences between the groups were statistically significant (*p* < 0.05), suggesting that young patients with concomitant PFO have a heavier burden of WMH (Table 2; Figure 2).

**Table 2:** Comparison of PVH and DWMH grades between the PFO group and control group.

Variables	PFO group (*n* = 120)	Control group (*n* = 121)	*Z/χ^2^*	*p-*value
PVH [*n* (%)]			35.740	<0.001
Grade 0	26 (21.7%)	63 (52.1%)		
Grade 1	61 (50.8%)	52 (42.9%)		
Grade 2	25 (20.8%)	6 (5.0%)		
Grade 3	8 (6.7%)	0 (0.0%)		
DWMH [*n* (%)]			101.424	<0.001
Grade 0	16 (13.3%)	87 (71.9%)		
Grade 1	50 (41.7%)	32 (26.4%)		
Grade 2	28 (23.3%)	2 (1.7%)		
Grade 3	26 (21.7%)	0 (0.0%)		

PVH, periventricular hyperintensity; DWMH, deep white matter hyperintensity; WMH, white matter hyperintensity.

Data are presented as *n* (%). The chi-square test was used for comparison of categorical variables between the two groups. Grade 0 indicates no WMH; Grade 3 indicates severe WMH. Control group: Individuals without PFO, matched for age and sex from the same clinical population.

### 3.3 Results of inter-rater reliability testing

To ensure the reliability of the radiological assessment results, two senior neurologists, who were blinded to the PFO status of the study participants, independently graded the periventricular white matter hyperintensities (PVH) and deep white matter hyperintensities (DWMH) in all enrolled participants using the Fazekas scale and conducted inter-rater reliability testing. The results showed that the weighted Kappa coefficient for PVH grading was 0.899 (*p* < 0.001), and for DWMH grading was 0.883 (*p* < 0.001). The PVH and DWMH grading results in this study were stable and reliable, with minimal measurement bias, and could be used for subsequent statistical analysis.

### 3.4 Partial correlation analysis of PFO-related indicators and WMH grading

After adjusting for confounding factors such as age and sex, the results of the partial correlation analysis showed that PFO diameter was significantly positively correlated with PVH (*r* = 0.398, *p* < 0.001) and DWMH (*r* = 0.671, *p* < 0.001); RLS grade was positively correlated with PVH (*r* = 0.319, *p* < 0.001) and DWMH (*r* = 0.609, *p* < 0.001); RLS type was positively correlated with PVH (*r* = 0.346, *p* < 0.001) and DWMH (*r* = 0.602, *p* < 0.001). Additionally, PVH was significantly positively correlated with DWMH (*r* = 0.531, *p* < 0.001), suggesting a high degree of consistency between the two white matter hyperintensity burdens; PFO diameter was significantly positively correlated with both RLS grade (*r* = 0.799, *p* < 0.001) and RLS type (*r* = 0.831, *p* < 0.001), suggesting that these indicators exhibit high consistency in reflecting shunt burden.

**Figure 3:**
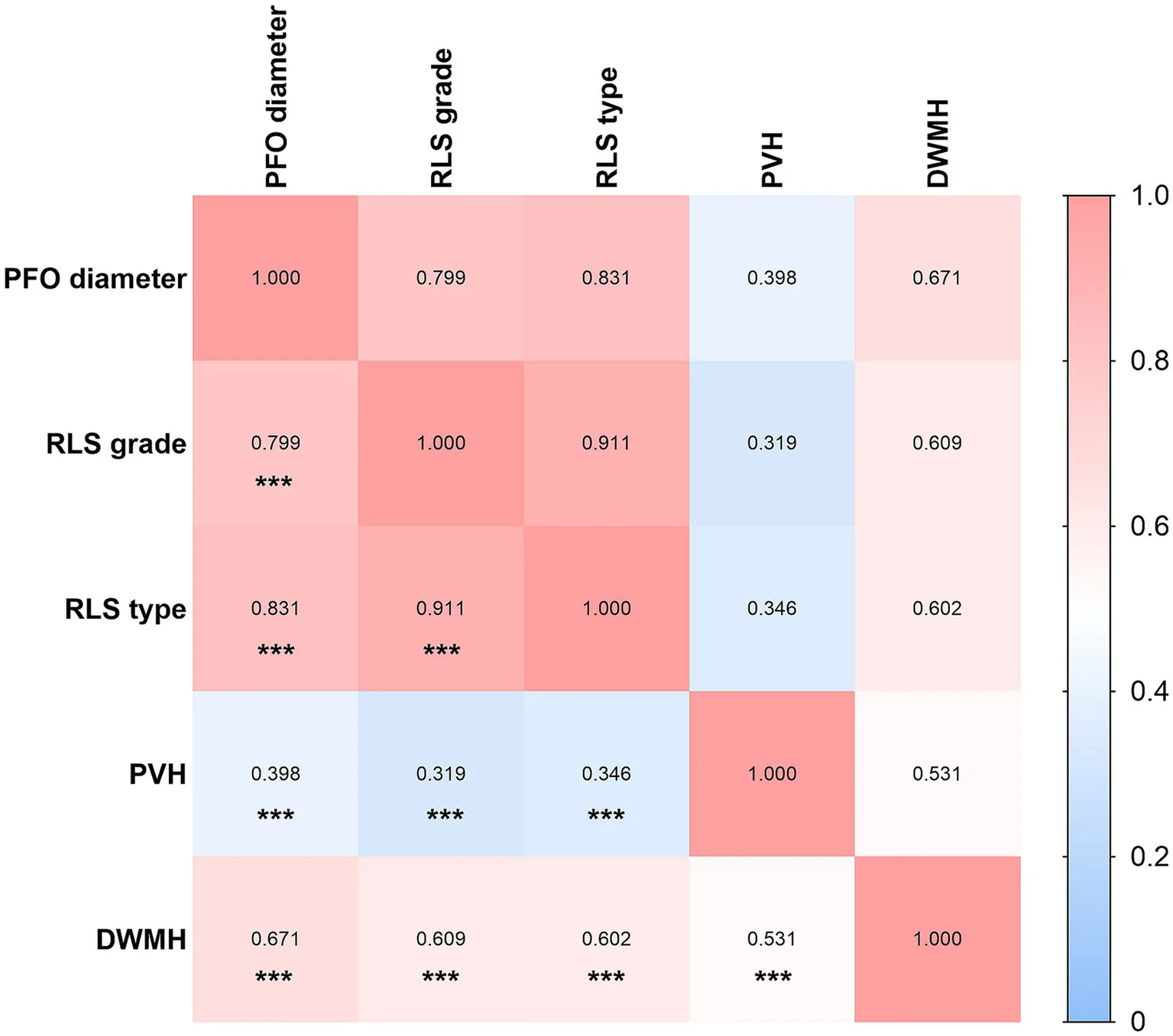
Heatmap of partial correlation coefficients between PFO-related indicators and WMH grades. After adjusting for confounding factors such as age and sex, the values in the figure represent the correlation coefficients between variables; the color gradient from blue to red indicates increasing correlation. PFO, patent foramen ovale; RLS, right-to-left shunt; PVH, periventricular hyperintensity; DWMH, deep white matter hyperintensity. ***p < 0.001.

Taken together, these results suggest that in young adults, a larger PFO diameter, higher RLS grade, and RLS subtype of persistent shunting are associated with a heavier burden of white matter hyperintensity lesions. Furthermore, the correlation between PFO-related indicators and DWMH is significantly stronger than that with PVH. The partial correlation coefficient matrix among the variables is shown in Figure 3.

### 3.5 Association between PFO-related indicators and WMH grading in young adults

To identify independent risk factors for WMH severity, we performed multinomial logistic regression analyses with PVH and DWMH grades as dependent variables, respectively. The parallel lines test was conducted to verify the suitability of the proportional odds model; as the test was significant for both PVH and DWMH (*p* < 0.05), indicating violation of the proportional odds assumption, we proceeded with multinomial logistic regression.

Considering the significant collinearity among PFO-related indicators (PFO diameter, RLS grade, and RLS type; correlation coefficients *r* = 0.799–0.831, *p* < 0.001, as shown in Figure 3), a forward stepwise selection approach (entry criterion *p* < 0.05, removal criterion *p* > 0.10) was applied to avoid multicollinearity and identify the most robust predictor(s). As a result, PFO diameter was the only variable retained in the final model.

**Figure 4:**
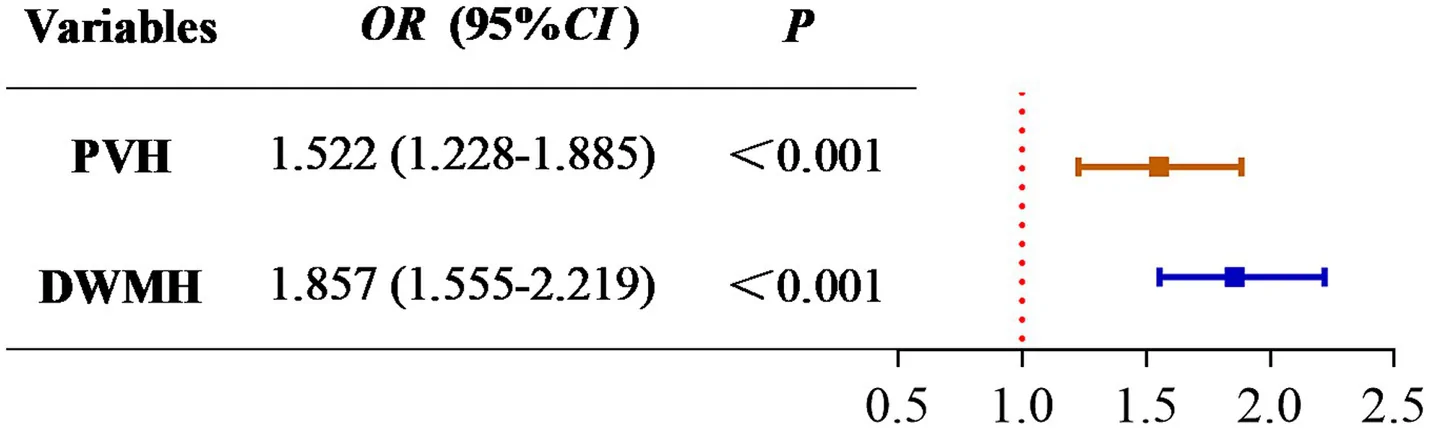
Forest plot of the multivariate-adjusted odds ratios (*ORs*) for severe white matter hyperintensities (Grade 3 vs. Grade 0) in young adults. The model was adjusted for age and sex. Squares represent point estimates of *ORs* for a 1-mm increase in PFO diameter, with horizontal lines indicating 95% confidence intervals (*CIs*). The vertical dashed line at *OR* = 1 indicates the null effect. PVH, periventricular hyperintensity; DWMH, deep white matter hyperintensity.

With Grade 0 (no WMH) as the reference group, the final model (adjusted for age and sex) revealed that PFO diameter was independently associated with both severe PVH (OR = 1.522, 95% CI: 1.228–1.885, *p* < 0.001) and severe DWMH (OR = 1.857, 95% CI: 1.555–2.219, *p* < 0.001) (Table 3; Figure 4). These results suggest that each 1-mm increase in PFO diameter may be associated with approximately a 1.5- to 1.9-fold increase in the odds of severe WMH in young adults.

**Table 3:** Multinomial logistic regression analysis of PFO diameter in relation to severe PVH and DWMH (Grade 3 vs. Grade 0) in young adults.

Variables	*OR*	95% CI		*p*-value
		Lower limit	Upper limit	
PVH	1.522	1.228	1.885	<0.001
DWMH	1.857	1.555	2.219	<0.001

OR, odds ratio; CI, confidence interval; PVH, periventricular hyperintensity; DWMH, deep white matter hyperintensity; PFO, patent foramen ovale.

Grade 0 (no WMH) was used as the reference group. Data represent the risk of severe WMH (Grade 3) compared with no WMH (Grade 0). The multinomial logistic regression model was used because the proportional odds assumption was violated (parallel lines test, both *p* < 0.05). The model was adjusted for age and sex. Due to significant multicollinearity among PFO diameter, RLS grade, and RLS type (correlation coefficients *r* = 0.799–0.831, all *p* < 0.001), a forward stepwise selection approach (entry criterion *p* < 0.05, removal criterion *p* > 0.10) was applied; PFO diameter was the only variable retained in the final model.

Age and sex were included as covariates in the model but were not the primary focus of this analysis. In the DWMH model, age showed a significant independent effect (OR = 1.271, 95% CI: 1.139–1.418, *p* < 0.001), whereas no significant age effect was observed in the PVH model (*p* = 0.242). Sex was not a significant predictor in either model (*p* > 0.2 for both).

## 4 Discussion

Using a multicenter retrospective case–control design, our study investigated the association between PFO and WMH in young adults and conducted a stratified analysis to identify independent risk factors for PVH and DWMH. The results showed that the WMH burden was significantly higher in the PFO group than in the control group. After adjusting for confounding factors, PFO diameter was associated with both PVH and DWMH severity, with a more pronounced effect on DWMH.

This study found that among young adults without hypertension, diabetes, or stenosis of large intracranial vessels, patients with PFO had a significantly greater WMH burden, suggesting that PFO may be associated with white matter changes independently of traditional vascular risk factors. Furthermore, young adults have relatively intact cerebral vascular structures and a relatively low burden of atherosclerosis (14). Therefore, this finding further highlights the role of PFO-related pathophysiological mechanisms in the development and progression of WMH (15). Previous studies have primarily focused on the association between PFO and cryptogenic stroke and migraine, with relatively limited exploration of the etiology of WMH in young adults without traditional vascular risk factors (16, 17). In this study, we identified PFO as a potential cause of WMH in young adults, a finding consistent with the conclusions of Li et al. (8). This suggests that even in young adults with PFO who exhibit no obvious clinical symptoms, latent white matter changes may still occur (18).

Furthermore, according to the results of the partial correlation analysis in this study, PFO diameter, RLS grading, and classification were all positively correlated with PVH and DWMH, with PFO diameter showing the strongest correlation with DWMH. From the perspective of anatomical and blood supply characteristics, the impact of PFO on DWMH was significantly stronger than that on PVH, which may be closely related to differences in blood supply to white matter regions (19). Specifically, the deep white matter is supplied by long penetrating arteries that are functionally end-arteries with limited collateral circulation, making this region particularly vulnerable to hemodynamic fluctuations and microembolic insults that may arise from right-to-left shunting; in contrast, periventricular white matter has a relatively richer anastomotic network and may be more resilient to such insults (20–22). However, we acknowledge that the DWMH-predominant pattern differs from the classic distribution of embolic lesions, and the exact mechanisms require further investigation. These findings are consistent with the hypothesis that PFO may contribute to white matter damage through potential mechanisms including paradoxical microembolism, cerebral hypoperfusion, and endothelial dysfunction, thereby promoting myelin loss and gliosis and ultimately leading to WMH, with damage to the deep white matter being particularly characteristic (23–25). However, the relative contribution of each mechanism cannot be determined from the current imaging data alone. This study also shows that age may have an independent effect only on DWMH, suggesting that age-related vascular degenerative changes may synergize with PFO-related damage to exacerbate deep white matter lesions (3). Multivariate logistic regression analysis further suggested that PFO may be associated with WMH in young adults, whereas neither the RLS grading nor the RLS classification demonstrated independent predictive value. This finding may be attributed to the significant multicollinearity between PFO diameter and RLS-related indicators, which was addressed using a stepwise regression approach. The stepwise selection process identified PFO diameter as the most stable and reliable predictor, suggesting that it may serve as a proxy for overall shunt burden in clinical risk assessment. Previous studies have demonstrated that PFO diameter, as an objective quantitative measure, directly determines the flow rate and stability of RLS (26). This study is highly consistent with the findings of Pan et al. (27), indicating that PFO diameter is a more stable and reliable radiological predictor of PFO-associated cerebral microvascular damage than RLS burden. This also provides new evidence for optimizing the clinical assessment system for PFO-associated cerebral vascular lesions. In light of recent research advances on PFO and cerebral small-vessel disease (28–30), our findings further suggest that when evaluating unexplained hyperintensities in the white matter of young adults, attention should be paid not only to traditional vascular risk factors but also to the potential influence of cardiac structural and shunt factors. Early completion of a cardiac ultrasound and measurement of PFO diameter are recommended to determine the presence of PFO-related brain damage, thereby enabling timely intervention.

This study has certain limitations. First, as a retrospective, multicenter study, our sample was relatively concentrated, which may introduce selection bias. Second, the analysis was limited to PFO diameter, RLS grading, and classification; it did not incorporate additional anatomical features such as atrial septal aneurysms or shunt morphology, nor did it include indicators such as cerebral perfusion, inflammatory factors, or coagulation parameters, resulting in an incomplete exploration of the underlying mechanisms. Third, the use of the Fazekas visual rating scale to assess WMH, while widely accepted and practical, involves a degree of subjectivity and may be less sensitive to mild lesion burdens, particularly in young adults with relatively low WMH volumes. Future studies could incorporate quantitative WMH volumetry, lesion count analysis, and lesion location mapping to provide more precise and objective assessments. Additionally, detailed evaluation of other CSVD markers such as lacunes, enlarged perivascular spaces, and cerebral microbleeds would help further characterize the nature of PFO-associated white matter changes. Fourth, our study did not include direct measurements of cerebral perfusion (e.g., arterial spin labeling, CT/MR perfusion, or transcranial Doppler flow parameters) or cerebrovascular reactivity. Therefore, the hypothesized role of chronic cerebral hypoperfusion in mediating the PFO-WMH association remains speculative and should be investigated in future studies with dedicated perfusion imaging. Fifth, as the primary objective of this study was to investigate WMH as a risk factor outcome rather than to comprehensively characterize all CSVD imaging features, we did not systematically evaluate additional MRI markers such as lacunes, enlarged perivascular spaces, cerebral microbleeds, or detailed lesion distribution patterns (e.g., cortical/juxtacortical involvement or posterior circulation involvement). Such data would have been valuable to further distinguish PFO-associated white matter changes from conventional small vessel disease. Future studies incorporating comprehensive CSVD imaging markers are warranted to better characterize the nature of PFO-associated brain injury. Sixth, information on migraine status, including the presence and subtype (with or without aura), was not systematically collected in this retrospective study. As migraine is a potential confounder in the association between PFO and WMH (8, 9, 31), we cannot exclude its influence on our findings. Future prospective studies should incorporate detailed migraine assessment.

## 5 Conclusion

This multicenter retrospective case–control analysis suggests that PFO may be associated with the presence and severity of WMH in young adults after excluding traditional vascular risk factors, with a more pronounced effect on DWMH. Age was associated only with the severity of DWMH, whereas RLS grading, RLS classification, and gender did not demonstrate independent predictive value. Compared with RLS burden, PFO diameter may serve as a useful radiological indicator for assessing PFO-related white matter changes in young adults and could inform etiological screening and risk stratification in cases of cryptogenic white matter lesions.

## Data Availability

The original contributions presented in the study are included in the article/supplementary material, further inquiries can be directed to the corresponding authors.
